# Could Gait Biomechanics Become a Marker of Atypical Neuronal Circuitry in Human Development?—The Example of Autism Spectrum Disorder

**DOI:** 10.3389/fbioe.2021.624522

**Published:** 2021-03-16

**Authors:** Marine Jequier Gygax, Anne M. Maillard, Julien Favre

**Affiliations:** ^1^Service des Troubles du Spectre de l’Autisme, Department of Psychiatry, Lausanne University Hospital, Lausanne, Switzerland; ^2^Swiss BioMotion Lab, Department of Musculoskeletal Medicine, Lausanne University Hospital and University of Lausanne (CHUV-UNIL), Lausanne, Switzerland

**Keywords:** autism, biomechanics, gait, neuronal circuit development, biomarker, walking, patterns, machine learning

## Abstract

This perspective paper presents converging recent knowledge in neurosciences (motor neurophysiology, neuroimaging and neuro cognition) and biomechanics to outline the relationships between maturing neuronal network, behavior, and gait in human development. Autism Spectrum Disorder (ASD) represents a particularly relevant neurodevelopmental disorder (NDD) to study these convergences, as an early life condition presenting with sensorimotor and social behavioral alterations. ASD diagnosis relies solely on behavioral criteria. The absence of biological marker in ASD is a main challenge, and hampers correlations between behavioral development and standardized data such as brain structure alterations, brain connectivity, or genetic profile. Gait, as a way to study motor system development, represents a well-studied, early life ability that can be characterized through standardized biomechanical analysis. Therefore, developmental gait biomechanics might appear as a possible motor phenotype and biomarker, solid enough to be correlated to neuronal network maturation, in normal and atypical developmental trajectories—like in ASD.

## Introduction

Although gait biomechanics during human development has been substantially studied, little is known about the neuronal correlates of this motor ability acquisition. As detailed by Dewolf et al. (2020b), emerging gait during early development requires successive interplays and activations of specific brain circuits. Therefore, one might wonder whether gait biomechanics could one day be seen as an expression of the maturation of brain functions.

A way to advance our knowledge in the field would be to study walking biomechanics in different developmental trajectories and correlate the biomechanical data with those assessing brain functions, such as neuroimaging and neuro-electrophysiology. In that perspective, as explained below, autism spectrum disorder (ASD) is a particularly interesting example of alternative brain development trajectory.

ASD is a neurodevelopmental disorder (NDD) related to subtle alterations in brain circuits and functions (Moyses-Oliveira et al., 2020; Shohat et al., 2020). These alterations result from genetics and epigenetics changes and might concern different cerebral regions and circuits (Esposito and Venuti, 2008). Despite current understanding, the diagnosis of ASD solely relies on the observation of clinical criteria according to the DSM-5 (Diagnostic and Statistical Manual of Mental Disorders, 5th edition) (Association, 2013; Lloyd et al., 2013). Particularly, core symptoms in ASD include deficits in social and communication skills, along with restricted interests and repetitive movements and actions. This fairly open set of behavioral criteria contribute to the high clinical heterogeneity in individuals receiving an ASD diagnosis. In spite of this heterogeneity, clinical scales, interview and age-adapted diagnostic tools allow ASD to be diagnosed in early development (18–24 months old). However, no reliable biological markers exist to support or confirm the clinical diagnosis of ASD, to establish a prognosis, or to follow the developmental trajectory. Furthermore, markers to distinguish different subgroups of ASD are still to be defined (Reichow et al., 2012; Studer et al., 2017). This absence of marker is clearly a major limitation to the advance of ASD research and therapeutic management.

Atypical sensory processing is highly prevalent in ASD (Ben-Sasson et al., 2019) and has been reported already in the first description of the disorder (Kanner, 1958b). Since then, ASD studies using clinical scores, questionnaires, and electrophysiology (Robertson and Baron-Cohen, 2017) have reported that the development of the sensory system is probably altered at different levels of processing, from sensory detection through multisensory integration. Alterations in sensory processing have notably been suggested to modify the perception of the environment and the way the individuals adapt their behavior with respect to the surroundings. Interestingly, during brain development, the sensory system matures “hand-in-hand” with the motor system, and sub-optimal functioning in one system has been shown to influence the other (Whyatt and Craig, 2013). A review on motor abilities in ASD using a computational approach suggested two possible origins of the atypical motor development: aberrant sensory noise and poor multisensory integration (Gowen and Hamilton, 2013). In this context, ASD appears particularly relevant to further explore the relationship between sensory and motor system developments. This idea is well supported by the literature review of Mosconi and Sweeney suggesting that sensory-motor dysfunction might be considered as primary features of ASD, assessable at a very young age, even before the behavioral core features of the diagnosis can be confirmed (Mosconi and Sweeney, 2015). In this prior review, the authors exposed how disrupted sensory-motor systems might participate to movement deficits in ASD and underlined the relevance to study motor control in this clinical population, with a possibility to use the motor signature to parse the clinical heterogeneity in ASD. Putting these considerations into practice points out to gait analysis. Indeed, walking is probably the most promising movement related to motor control to analyze for that purpose, as it represents the primary means of locomotion throughout human life, a major developmental milestone and a factor in social construct that can be described by standardized biomechanical measures.

Through a literature analysis, this paper aims at setting the basis for further research on the variability of motor control development, with the perspective to consider gait biomechanics as an expression of brain functions in different developmental trajectories. To illustrate our purpose, we use ASD as an example of atypical development.

## Motor Deficit in ASD

Alike sensory atypicalities, motor deficits were already reported in the first description of ASD, more than half a century ago (Kanner, 1958a,b). In recent years, different forms of motor impairment have been reported in about 80% of the school-aged children and adults diagnosed with ASD, mostly by case series or cross-sectional studies. Specifically, postural control deficit (Lim et al., 2017), stereotypies (purposeless repetitive movements) (Goldman et al., 2009; Uljarevic et al., 2017), clumsiness, coordination and manual praxis disorders (Weber, 1978; Barrow et al., 2011; Fournier et al., 2014; Kaur et al., 2018), increased joint mobility associated with hypotonia, and gait abnormalities (Shetreat-Klein et al., 2014), as young as 6 months after independent walking (Esposito and Venuti, 2008), have been observed in ASD. Furthermore, hypotonia, a characteristic that can be observed as early as 4–6 months of age, has been associated with autistic traits at 6 years in a longitudinal study (Serdarevic et al., 2017). Additionally, early gross motor developmental disorders have been associated with later ASD diagnosis and social communication deficits (Lloyd et al., 2013; LeBarton and Iverson, 2016). These consistent observations have even motivated some authors to claim that atypical neuro-motor development should be considered as “a putative endophenotype for ASD” (Esposito and Pasca, 2013), or at least as a core feature of the disorder (Teitelbaum et al., 1998; Rinehart et al., 2006; Dufek et al., 2017). Although it is clear that the motor system is altered in ASD, no specific developmental motor pattern has yet been identified, with the possible exception of the presence of dyspraxia in young adults (Dziuk et al., 2007; Kaur et al., 2018). Nevertheless, the recurrent observations of atypical motor development in ASD strongly support that more focused and objective assessments of the movement could highlight “bio-behavioral marker” (Anzulewicz et al., 2016). This possibility is particularly supported by prior works showing differences in upper limb kinematics (Crippa et al., 2015) and in micro-movements variability (Torres, 2013; Torres et al., 2013) between ASD and typically developing (TD) subjects.

Independent walking requires numerous processes like neurological development, musculo-skeletal maturation and experience accumulation, in order to be achieved between 12 and 18 months of age (Burnett and Johnson, 1971a,b; Forssberg, 1985; Thelen and Cooke, 1987; Ivanenko et al., 2007). Analyzing gait biomechanics seems particularly relevant to better understand ASD, and hopefully better manage this disorder in the future, because walking is a robust milestone of the neuro-motor system maturation. Furthermore, gait is a movement that can be continuously observed lifelong. In that aspect, it could represent an indicator of the brain sensory-motor functions across development and provide an individual motor “signature” (Mosconi and Sweeney, 2015). An additional motivation to focus on walking comes from the tremendous knowhow in quantitative gait analysis, with well-established methods and prior uses in infants and children affected by different developmental disorders, like prematurity (Cahill-Rowley and Rose, 2016), cerebral palsy (Galli et al., 2010) and genetic syndrome (Naito et al., 2015), in addition to ASD, as detailed in the following paragraph.

**FIGURE 1:**
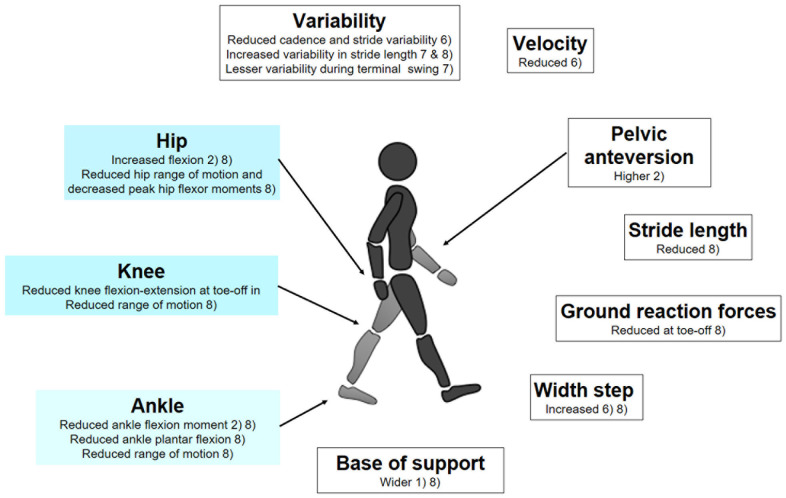
Gait biomechanics data found in children with ASD, in comparison to control groups, matched for age and gender; (1) Manicolo et al. (2019); (2) Biffi et al. (2018); (3) Hasan et al. (2017); (4) Eggleston et al. (2017); (5) Dufek et al. (2017); (6) Lim et al. (2016)—CV Coefficient of variation; (7) Eggleston et al. (2020) (8) Kindregan et al. (2015)—review of 11 studies assessing gait biomechanics in children with ASD.

In 2015, Kindregan et al. (2015), conducted a review on walking biomechanics in children with ASD and concluded to a more unstable gait in patients affected by ASD. Since the publication of the review by Kindregan et al., other studies analyzed gait biomechanics in children with ASD (Lim et al., 2016; Dufek et al., 2017; Eggleston et al., 2017, 2020; Hasan et al., 2017; Biffi et al., 2018; Manicolo et al., 2019). The results from these works and from the studies in the review by Kindregan et al. are summarized in Figure 1.

Unfortunately, while consistent in the observations of altered gait biomechanics in ASD, important methodological differences among studies prohibited the identification of specific biomechanical markers. This might come from limitations of prior gait studies displaying relatively small sample sizes and broad age-range in the cross-sectional studied cohorts, not taking into account the variability of gait during development (Hallemans et al., 2006). Furthermore, confounding factors such as ASD severity, attention deficit hyperactivity disorder (ADHD) comorbidity, medication, and obesity were most of the time not addressed, despite ASD movement patterns repeatedly reported as heterogeneous (Calhoun et al., 2011; Dufek et al., 2017; Eggleston et al., 2017). Nevertheless, gait biomechanics should not be viewed as a dead-end, but rather as a point in ASD research where a step toward large-scale studies has to be taken to allow compensating for the natural inter-individual variability, across ASD diagnosis and developmental trajectories. Recent results with other medical conditions have shown that applying machine learning methods on adequately-sized datasets could identify relevant gait patterns (Kwon et al., 2020), even when prior studies with smaller sample sizes reported rather inconsistent results (Mills et al., 2013; Favre and Jolles, 2016). Therefore, advanced statistical modeling or machine learning approaches could also certainly identify gait patterns specific to ASD. For instance, a statistical framework analysis applied to micro-movements during gait allowed distinguishing subjects with genetic syndrome, from subjects with ASD and controls (Torres et al., 2016). While identifying ASD-specific patterns will indisputably constitute a significant advance, it could be possible to go further by considering, not only the gait data, but also their relationships with complementary measures. For example, analyzing the associations between the structure of the knee joint and gait biomechanics offered interesting perspectives in the understanding of degenerative joint disease (Edd et al., 2018). In the case of ASD, it could be particularly valuable to bridge biomechanical and neural circuitry data, such as neuroimaging and electrophysiology.

## Gait Biomechanics and Brain Networks Maturation

In ASD, there are many brain structures thought to be involved in the behavioral semiology, and interestingly, many of these identified structures play a role in the motor system. Since more than a decade, the medial prefrontal cortex, has been recognized as “hub” central nervous system structure implicated in the semiology of autism at the integrative level of functional domains such as perception-sensation and motor skills (Shalom, 2009). Connections between the medial prefrontal cortex and basal ganglia support initiation and inhibition of voluntary movements (Canales and Graybiel, 2000) and are serving higher order cognitive functions in addition to motor control (Leisman et al., 2014). The basal ganglia are implicated not only in neural circuitry connecting sub-regions of prefrontal cortex, but also specific regions in cerebellum. The different neural systems known to present with modified structures and functions in ASD are also modulating afferent and efferent neural information at the level of the pons and the brain stem (for a review see Mosconi and Sweeney, 2015). The next paragraphs point out the role played in motor systems and social cognition by two brain structures – cerebellum and superior temporal sulcus (STS) and one well-described brain network, the default mode network (DMN).

Cerebellar structures and functions differ in ASD children and adult (for a review see Cook et al., 2013). Cerebellum is involved in movement control and in gait maturation, however, it is probably equivalently involved in emotion regulation and social cognition. In an extensive review on cerebellum, Van Overwalle et al. (2020) detailed how this brain structure is a core hub of brain networks involved in motor control and social cognition. It possesses important connections with cortical structure like the right superior temporal sulcus (STS) (Sokolov et al., 2014) involved in face and language processing in social context (Patel et al., 2019; Sato and Uono, 2019). In mouse models of ASD, manipulating circuitry linking prefrontal cortex to cerebellum allowed modulating repetitive motor behavior and social interest (Kelly et al., 2020). In adults with ASD, recognition of biological motion is preserved, but activation of STS during this task is not identical to controls (Alaerts et al., 2017), suggesting a variant recruitment of cerebral networks. An intact neural motor system is mandatory for perception-action coupling, as well as social understanding of the motor intentions, interpreted as visuo-motor resonance (Becchio and Castiello, 2012). These cognitive and somatosensory functions participate to the maturation of the body map representation already in infancy (Marshall and Meltzoff, 2020), and to reciprocal social cognition (Cook, 2016), which could not deploy without reliable sensory-motor system functioning.

Ontogenetically, gait is specific to human development (Forssberg, 1985). During gait development, spinal neural networks (Central Pattern Generators—CPG) are progressively modulated by supra-spinal brain structures. Spinal neural networks have mostly been studied in animal models, and their maturation requires the interplay of speed-dependant spinal interneurons (Deska-Gauthier et al., 2020) and activation/inactivation of sets of motor neurons (Ausborn et al., 2018). A detailed overview of the known and putative mechanisms is provided by Dewolf et al. (2020b). It shows also how little is known about the maturation of the cortical and sub-cortical brain structures leading to adult gait patterns, the timing of their activation, and their functions in this process (Petersen et al., 2010; Dewolf et al., 2020a,b). A recent human study suggested that functional connectivity maturation of the Default Mode Network (DMN) and related motor networks are correlated with walking skills at the age of 12 months. Walking skills was assessed by developmental clinical scale (including data about walking). This work also showed the progressive involvement of additional networks supporting the motor development, revealing possible neural mechanisms linking an early life motor behavior -the start of independent walking—to brain circuitry (Marrus et al., 2018). Interestingly, DMN is recognized for its role in self-referential processing (Raichle, 2015). Altered connectivity of the DMN and motor network have also been correlated to social deficits (Yerys et al., 2015; Nebel et al., 2016). Functional connectivity has been correlated to motor development in children born very preterm with NDD and brain structure maturation and motor skills have been shown to differ in these children compared to those born at term (Wheelock et al., 2018). To our knowledge, no such longitudinal studies combining motor system assessment and functional connectivity has been performed in a cohort of children with ASD.

To summarize, circuitry serving gait maturation and movement control in infants and toddlers most probably participates later to skills like language and higher cognitive function (Leisman et al., 2014), and could also represent a neuro-anatomical substrate to social cognition. Neuroimaging studies in cognitive development might gain from correlating data of standardized biomechanical assessment, like gait biomechanics, sensory-motor profiles and behavioral assessment. This might not only improve the understanding of the “neurophysiological signature” in ASD (Mosconi and Sweeney, 2015), but also help uncover neural substrate dedicated to gait maturation.

## Perspectives

In order to use gait biomechanics as a marker of atypical development, it seems research should first focus on longitudinal studies assessing gait biomechanics in large datasets of typically developing children to establish normative data about motor phenotypes. It will require specific data analysis like dedicated statistical frameworks and machine learning methods. Improving our knowledge on typically developing children would allow for comparison of motor development in ASD and other NDD.

Based on previous data, the developmental time-window, related to chronological age or developmental and cognitive milestones, might be crucial to identify specific movement patterns and related biomechanical characteristics in clinical research. As an early motor emerging skill, independent gait represents one of these specific developmental time-window. During the first months of independent walking, the maturation of kinetic and kinematic characteristics have been described, and might evolve progressively to an adult gait pattern (Hallemans et al., 2006; Ivanenko et al., 2007; Lacquaniti et al., 2012a). At early stage of development, gait is still not influenced by executive function, that are developing later and that are modulated by cultural environment (Cook, 2016). Additionally, gait represents a lifelong motor skill, easily reproducible and repetitively assessable, from very early life, to adulthood.

As seen, the maturation of independent walking requires the modulation of spinal neuronal networks by the cortico-spinal control. This could not be achieved without a high flexibility of the systems (Lacquaniti et al., 2012b). In ASD population, fragile sensory integration and limited flexibility might generate altered gait maturation in a specific way. ASD is a frequent neurodevelopmental lifelong condition, diagnosed very early in life.

**FIGURE 2:**
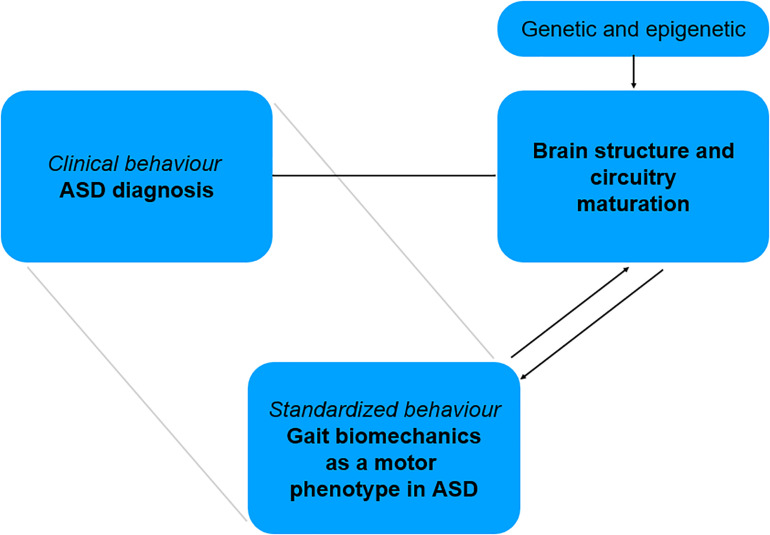
Bridging motor phenotype in ASD to related brain structure and circuitry involved in motor system maturation.

For these different reasons, ASD represents a particularly relevant condition to study the relationships between gait biomechanics and brain circuits. Instrumented gait analysis could offer a possibility to obtain early quantitative data of affected motor patterns and might help identify early motor system traits specific to this disorder. As a standardized data of a motor phenotype, gait biomechanics might be correlated with brain structure and circuitry changes. In this perspective, the comparison of two different developmental trajectories—ASD vs. typically developing children – through biomechanics and brain connectivity, might offer the potential to bridge in a specific manner a clinical diagnosis relying on behavioral criteria, to brain structure and network maturation (Figure 2). It might also help further uncover the changes in neural mechanisms implicated in independent walking across development. Such approach is in line with recent concepts of research in mental disease and NDD, which promote the identification of behaviors that can be standardized, with a focus on “circuit-behavior relationships” (RDoc Research Domain Criteria; for details see Cuthbert, 2014; Mittal and Wakschlag, 2017).

To achieve this, gait biomechanics might be considered an estimation of brain functions during development. It should be integrated in clinical research study design combining multi-modal investigations, including neuroimaging, neurophysiology and behavioral assessment of ASD. Due to the interrelated nature of sensory and motor systems maturation, modifying the sensory environment during gait assessment might influence biomechanics in a way to uncover the flexibility of the sensory-motor system, essential to gait maturation. Eventually, if gait biomechanics could be identified as a motor biomarker of ASD, it will add criteria to distinguish population of patients, and orientate follow-up and therapies dedicated to enhancing sensory-motor functions. By considering the model of ASD as first step to extend our knowledge about using biomechanics as an expression of brain function, these paradigms could certainly be generalized to other NDD or diseases and offer new research perspectives.

## Data Availability Statement

The original contributions presented in the study are included in the article/supplementary material, further inquiries can be directed to the corresponding author/s.

## Author Contributions

MJ designed and wrote the article. AM participated to the writing. JF contributed to the design and participated to the writing. All authors contributed to the article and approved the submitted version.

## Conflict of Interest

The authors declare that the research was conducted in the absence of any commercial or financial relationships that could be construed as a potential conflict of interest.
